# Transcriptome Analysis of Blunt Snout Bream (*Megalobrama amblycephala*) Reveals Putative Differential Expression Genes Related to Growth and Hypoxia

**DOI:** 10.1371/journal.pone.0142801

**Published:** 2015-11-10

**Authors:** Fu-Gui Li, Jie Chen, Xia-Yun Jiang, Shu-Ming Zou

**Affiliations:** Key Laboratory of Freshwater Aquatic Genetic Resources, Shanghai Ocean University, Huchenghuan Road 999, Shanghai, 201306, China; Temasek Life Sciences Laboratory, SINGAPORE

## Abstract

The blunt snout bream (*Megalobrama amblycephala*) is an important freshwater aquaculture species, but it is sensitive to hypoxia. No transcriptome data related to growth and hypoxia response are available for this species. In this study, we performed *de novo* transcriptome sequencing for the liver and gills of the fast-growth family and slow-growth family derived from ‘Pujiang No.1’ F10 blunt snout bream that were under hypoxic stress and normoxia, respectively. The fish were divided into the following 4 groups: fast-growth family under hypoxic stress, FH; slow-growth family under hypoxic stress, SH; fast-growth family under normoxia, FN; and slow-growth family under normoxia, SN. A total of 185 million high-quality reads were obtained from the normalized cDNA of the pooled samples, which were assembled into 465,582 contigs and 237,172 transcripts. A total of 31,338 transcripts from the same locus (unigenes) were annotated and assigned to 104 functional groups, and 23,103 unigenes were classified into seven main categories, including 45 secondary KEGG pathways. A total of 22,255 (71%) known putative unigenes were found to be shared across the genomes of five model fish species and mammals, and a substantial number (9.4%) of potentially novel genes were identified. When 6,639 unigenes were used in the analysis of differential expression (DE) genes, the number of putative DE genes related to growth pathways in FH, SH, SN and FN was 159, 118, 92 and 65 in both the liver and gills, respectively, and the number of DE genes related to hypoxic response was 57, 33, 23 and 21 in FH, FN, SH and SN, respectively. Our results suggest that growth performance of the fast-growth family should be due to complex mutual gene regulatory mechanisms of these putative DE genes between growth and hypoxia.

## Introduction

The blunt snout bream (*Megalobrama amblycephala*), also known as the Wuchang bream, is an herbivorous freshwater fish species with a high economic value in China. It is an endemic species, with its natural distribution restricted to the affiliated lakes of the Yangtze River, such as Liangzi Lake, Poyang Lake, and Yuni Lake [1–3]. The blunt snout bream is widely consumed as a delicacy and has been recognized as one of the main aquaculture species in the freshwater polyculture system of China since the 1960s [4]. As a consequence of selective breeding since 1984, the growth rate of ‘Pujiang No.1,’ a good breed of the blunt snout bream (F6), was increased by 29% in 1999 [5]. In 2011, the aquaculture industry of the blunt snout bream developed greatly, with a total production of 677,887 tons [6]. However, the blunt snout bream is a hypoxia-sensitive species, and a short period (<2 h) of hypoxia (less than 0.5 mg O_2_·L^−1^) at room temperature can be lethal [7]. Therefore, studying the inhibition mechanism of hypoxia on growth may be required for the genetic breeding of this fish species in the future.

Developing genome resources facilitates both structural and functional analyses of genes related to growth and hypoxic response in fish [8–14]. However, expressed sequence tags generated with the aim of identifying gene expression profiles related to growth and hypoxic response are not yet available for the blunt snout bream. In the present study, we used Illumina Hiseq ^™^ 2000 [15–19] sequencing to characterize the transcriptomes of eight samples, in order to provide the most comprehensive gene sequence resources for the blunt snout bream and to obtain differential expression (DE) genes related to growth and tolerance to hypoxia. We believe that these resources would greatly aid breeding programs and whole-genome association studies for the blunt snout bream.

## Materials and Methods

### Ethics statement

This study was approved by the institutional review board or ethics committee of Shanghai Ocean University (Permit Number: 2013016). All experiments were carried out in strict accordance with the guidelines on the care and use of animals for scientific purposes set by the Institutional Animal Care and Use Committee (IACUC) of Shanghai Ocean University, Shanghai, China.

### Experimental specimens

Blunt snout bream specimens were obtained from the Bream Genetics and Breeding Center (BGBC) of Shanghai Ocean University, Shanghai, China. Specimens that belonged to the fast-growth family (F) and slow-growth family (S) derived from the ‘Pujiang No.1’ F10 breed were used. In the breeding season of 2013, fertilized eggs were generated by artificial insemination, and 300 hatched larva from F and 300 from S were divided into 2 groups, respectively and cultured in four 16-m^2^ concrete ponds under hypoxic stress (~3 mg O_2_·L^−1^) or normoxia (~7 mg O_2_·L^−1^): fast-growth family under hypoxic stress, FH; slow-growth family under hypoxic stress, SH; fast-growth family under normoxia, FN; and slow-growth family under normoxia, SN. One-tenth of the water was replaced daily. Throughout the experimental period, the fish were fed daily (9:00 AM) to satiation by using a commercial feed. After 120 days of cultivation, the growth rate was measured (mean body weight of FH, SH, FN, and SN groups was 15.68 g, 8.43 g, 28.33 g, and 17.38 g, respectively; Table 1), and liver and gills from 3 random individuals of each group were used as samples.

**Table 1:** The detailed sample information of blunt snout bream.

Groups	Hypoxia treatment	No. of fish survived at 120-day	Final body weight (mean±s.d.)(g)	No. of fish sampled	Tissues sampled
Fast-growth family under normoxia (FN)	~7.0 mg/ L O_2_, from larvae to 120-day	142	28.33±0.29 ***	3	Liver and gills
Fast-growth family under hypoxia (FH)	~3.0 mg/ L O_2_, from larvae to 120-day	141	15.68±0.11 **	3	Liver and gills
Slow-growth family under normoxia (SN)	~7.0 mg/ L O_2_, from larvae to 120-day	141	17.38±0.32 **	3	Liver and gills
Slow-growth family under hypoxia (SH)	~3.0 mg/ L O2, from larvae to 120-day	139	8.43±0.07	3	Liver and gills

**p<0.01;

***p<0.001.

### RNA isolation

The fish were euthanized with 100 mg/L of MS-222 (tricaine methanesulfonate; Sigma, St. Louis, MO, USA) and maintained on ice before tissue collection. Liver and gills were collected, immediately frozen in liquid nitrogen, and stored at -80°C until RNA extraction. Total RNA was extracted using TRIzol reagent (Invitrogen, Carlsbad, CA, USA), according to the manufacturer’s instructions. RNA quality was checked using agarose gel electrophoresis and spectrophotometry. High-quality RNA with 28S:18S more than 1.5 and absorbance ratios OD_260_/OD_280_ = 1.8–2.2 and OD_260_/OD_230_ ≥ 2.0 was used for library construction and sequencing. Total RNA from each sample was standardized to 200 ng/μL. Equal volumes of total RNA of liver and gills from three individuals of the same group (FH, SH, FN, and SN) were combined into one pool, resulting in a total of eight RNA pools. The pools were treated with Turbo DNA-free (Ambion, Austin, TX, USA) and purified using the RNeasy Mini Kit (Qiagen, Valencia, CA, USA), according the manufacturer’s instructions. RNA quality and quantity were again determined at the end of the process.

### cDNA library construction and sequencing

Library construction was performed using Illumina Hiseq^™^ 2000 (Illumina, San Diego, CA, USA), according to the manufacturer’s instructions. Magnetic beads with oligo-dT were used to combine the poly-A of the mRNA for purifying the mRNA from the total RNA. The mRNA was then mixed with fragmentation buffer to obtain short fragments of about 155 bp. The fragments were used to synthesize first-strand cDNA with random primers, and first-strand cDNA was transformed into double-strand cDNA by using RNase H and DNA polymerase I. A paired-end library was constructed from the cDNA synthesized using the Genomic Sample Prep Kit (Illumina, San Diego, CA, USA). Fragments of desirable lengths (~155 bp) were purified using the QIAquick PCR Extraction Kit (Qiagen, Valencia, CA, USA), end-repaired, and linked with sequencing adapters. AMPure XP beads (Beckman Coulter, Shanghai, China) were used to remove unsuitable fragments, and the sequencing library was constructed using polymerase chain reaction (PCR). The multiplexed cDNA libraries were checked using PicoGreen (Quantifluor^™^-ST fluorometerE6090, Promega, CA, USA) and fluorospectrophotometry (Quant-iT PicoGreen dsDNA Assay Kit; Invitrogen, P7589) and quantified with Agilent 2100 (Agilent 2100 Bioanalyzer, Agilent, 2100; Agilent High Sensitivity DNA Kit, Agilent, 5067–4626), and the synthesized eight cDNA libraries were normalized to a 10 nM. Then, the sequencing library was gradually diluted and quantified to 4–5 pM and sequenced using the Illumina HiSeq^™^ 2000 platform (Shanghai Personal Biotechnology, Shanghai, China).

### Data filtering and de novo assembly

The clean data from all 8 transcriptomes were put together to do the assemble because this approach tend to get unigenes more accurate and comprehensive for samples from same species without genome-wide reference [20]. The adaptor contamination was removed, the reads were screened from 3′ to 5′ to trim the bases with a quality score of Q < 20 by using 5-bp windows, and the reads with a final length of less than 25 bp were removed. We analyzed the quality of data filtering by using FastQC (http://www.bioinformatics.babraham.ac.uk/projects/fastqc/). *De novo* transcriptome assembly was performed using Trinity (http://trinityrnaseq.sf.net) [20]. A K-mer library was constructed with the filtered reads, and the contigs were formed using Inchworm. Using Chrysalis, a component was built with the contigs, and de Bruijn graphs were generated. Then, Butterfly was used to optimize the de Bruijn graphs and create the final transcript through paths [21]. Transcripts with no reads mapped in all eight samples were considered as errors and removed. All the transcripts were searched against the blunt snout bream database, and those with no hits were then searched in the NCBI non-redundant (NR) database (http://ftp.ncbi.nlm.nih.gov/blast/db/) with the BLAST program (http://www.ncbi.nlm.nih.gov/); the transcripts from the same locus with the maximum hits were selected as unigenes. The software Get ORF [22] was used to predict the open reading frames of unigenes that could not be aligned to the databases in order to ascertain their sequence directions, with default settings, except for the parameter “–find,” being set as 1.

### Gene annotation and analysis

Blast2 GO program [23–26] was used to annotate the unigenes on the basis of GO terms related to the blunt snout bream and NR database annotation. Conservation of gene identities of the blunt snout bream and those of other species (zebrafish, medaka, *Tetraodon*, fugu, stickleback, human, mouse, and chicken) was analyzed using BLASTX. To annotate genes with common denominators or functional categories, the unigenes were also aligned to the eggNOG database (http://www.ncbi.nlm.nih.gov/COG/, http://eggnog.embl.de/version_3.0/). To summarize the pathway information, the KEGG database was used to perform pathway annotation (http://www.genome.jp/kegg/). We identified the sequences related to growth and low oxygen resistance pathways by referring to previous studies [27–36].

### Comparative expression analysis

DESeq was used to identify DE genes (http://www.huber.embl.de/users/anders/DESeq). Those fold change <0.5 or >2 with a *p*-value < 0.05 were considered as significant differential expression [37–40]. Volcano Plot was used to intuitively display the comparative expressions of unigenes. We performed cluster analysis of gene expression patterns by using Cluster 3.0 and TreeView (http://bonsai.hgc.jp/~mdehoon/software/cluster/software.htm/ and http://jtreeview.sourceforge.net) [41], MeV [42], and Java TreeView software packages [43]. DE genes between the liver and gills were screened, and GO enrichment analysis was performed using Blast2GO (http://www.blast2go.com/). The *p*-value indicated the degree of difference in expression. By compared with the entire genome database of other teleost fish, we acquired GO classification information of possible enrichment in function in the samples. The expression levels of up- or down-regulated DE genes were annotated using KO analysis. Location of DE genes in various pathways can be revealed using KEGG pathways (http://www.genome.jp/kegg/tool/map_pathway2.html). On the basis of the *p*-value, pathway classification enrichment analysis of DE genes was used to highlight the differences between the genes.

### Quantitative real-time PCR

Genes identified with the transcriptome sequencing analysis were validated and quantified using quantitative real-time (qRT)-PCR. Primers (Table 2) were designed according to Illumina sequencing data by using Primer Premier 5 (Premier, Canada)[40]. The stable housekeeping gene *β*-*actin* was used as the control [44]. Total RNA was obtained from the same samples used for Illumina sequencing. Reversed cDNA was synthesized using the PrimeScript^™^ RT reagent Kit with gDNA Eraser (Takara, Shanghai, China). qRT-PCR was performed using the CFX96 Touch^™^ real-time PCR Detection System (BioRad, USA), according to the manufacturer’s instructions. The reaction was conducted using a total volume of 20 μL containing 10 μL of SYBR Green Master Mix (Takara, Shanghai, China), 1 μL of diluted cDNA mix, 0.6 mL of each primer (10 mM), and 7.8 μL of RNase-free water. The thermal profile for SYBR Green RT-PCR was 95°C for 20 s, followed by 40 cycles of 95°C for 5 s, 58.5°C for 30 s, and 72°C for 30 s. Amplification and detection of only one PCR product were confirmed using melting curve analysis of the amplification products at the end of each PCR. All experiments were performed in triplicate. Expression levels of different genes were analyzed using the comparative CT method (2^-ΔΔCT^ method) [45].

**Table 2:** Genes and specific primers used for quantitative real-time PCR.

Gene name	Primer name	Primer sequence (5’-3’)
*β-actin*	β-actin-F, β-actin-R	CGTGCTGTTTTCCCTTCCATT, CAATACCGTGCTCAAAGGATACTT
*HIF-1α*	HIF-1α-F, HIF-1α-R	ATCACCTCACCAAGACACATCACA, TCTCCACCCACACAAAACCACC
*HIF-2α*	HIF-2α-F, HIF-2α-R	GGCTTCATTACCGTGGTTACAT, GTTCAGCTCCTTGCCTTTCTTT
*VEGF-A*	VEGF-A-F, VEGF-A-R	CCACGGAAACTGTTACAACG, CTTATCCATTCTGCGTCCCT
*EPO*	EPO-F, EPO-R	AGAGGAGCAAGCTCAAGAGG, TGGCATCTATGTGGGACTGT

## Results and Discussion

### Sequencing and data analysis

All eight raw reads have been deposited in the NCBI SRA database (accession number, SRP050593). RNA sequencing produced a total of 209 million 100-bp paired-end reads with an average of 26 million reads for each of the eight samples (S1 Table). Cleaning and quality checks for the raw data were performed and then put together to do the assemble. A total of 185 million trimmed reads were obtained with a useful data percentage of 85.67–86.51%, and the average length of each paired read was 195 bp (S2 Table). An assembly of the reads generated 465,582 contigs with a mean size of 335 bp and N50 of 536 bp for about 40.83%, and 237,172 transcripts were produced with an average length of 1,137 bp. A total of 31,338 unigenes were generated, with an average length of 2,050 bp and sizes ranging from 200 bp to 28,154 bp (Table 3). About 61.3% of the contigs were distributed in the 100–199-bp region and 90.2% were <599 bp, while 56.3% of the transcripts were <599 bp and 94% of the unigenes were 200–4,999 bp in size (S1 Fig).

**Table 3:** Statistical summary of cDNA sequences of the blunt snout bream by the Illumina Hiseq platform.

	Contigs	Transcripts	Unigenes
Total length (bp)	155,856,123	269,656,769	64,254,778
Sequence No.	465,582	237,172	31,338
Max Length (bp)	30,535	28,154	28,154
Average Length (bp)	335	1,137	2,050
N50	536	2,426	2,951
N50 Reads No.	51,666	32,051	6,974
GC%	40.83%	43.64%	45.65%

**Fig 1:**
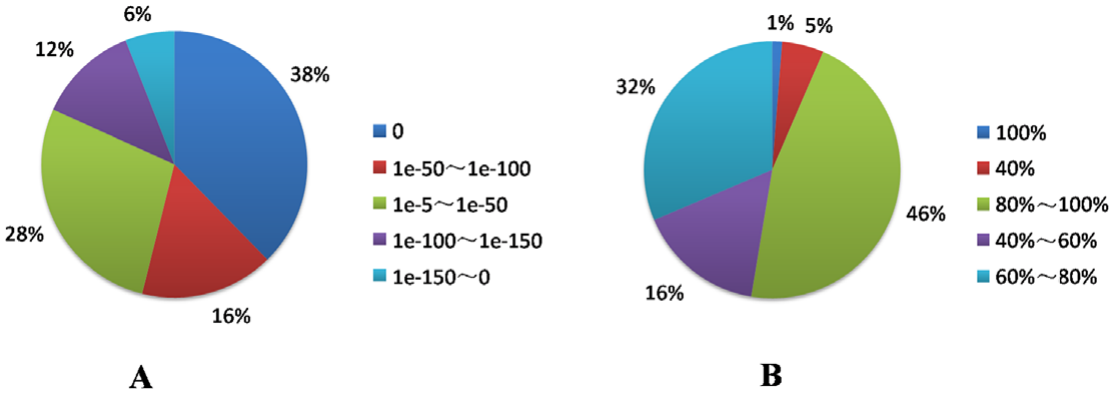
Landscape of unigene distribution in the blunt snout bream. (a) E-value distribution of unigenes searched against public databases with an E-value cut-off of 1E-50. (b) Identity distribution of unigenes searched against public databases with an E-value cut-off of 1E-50.

**Fig 2:**
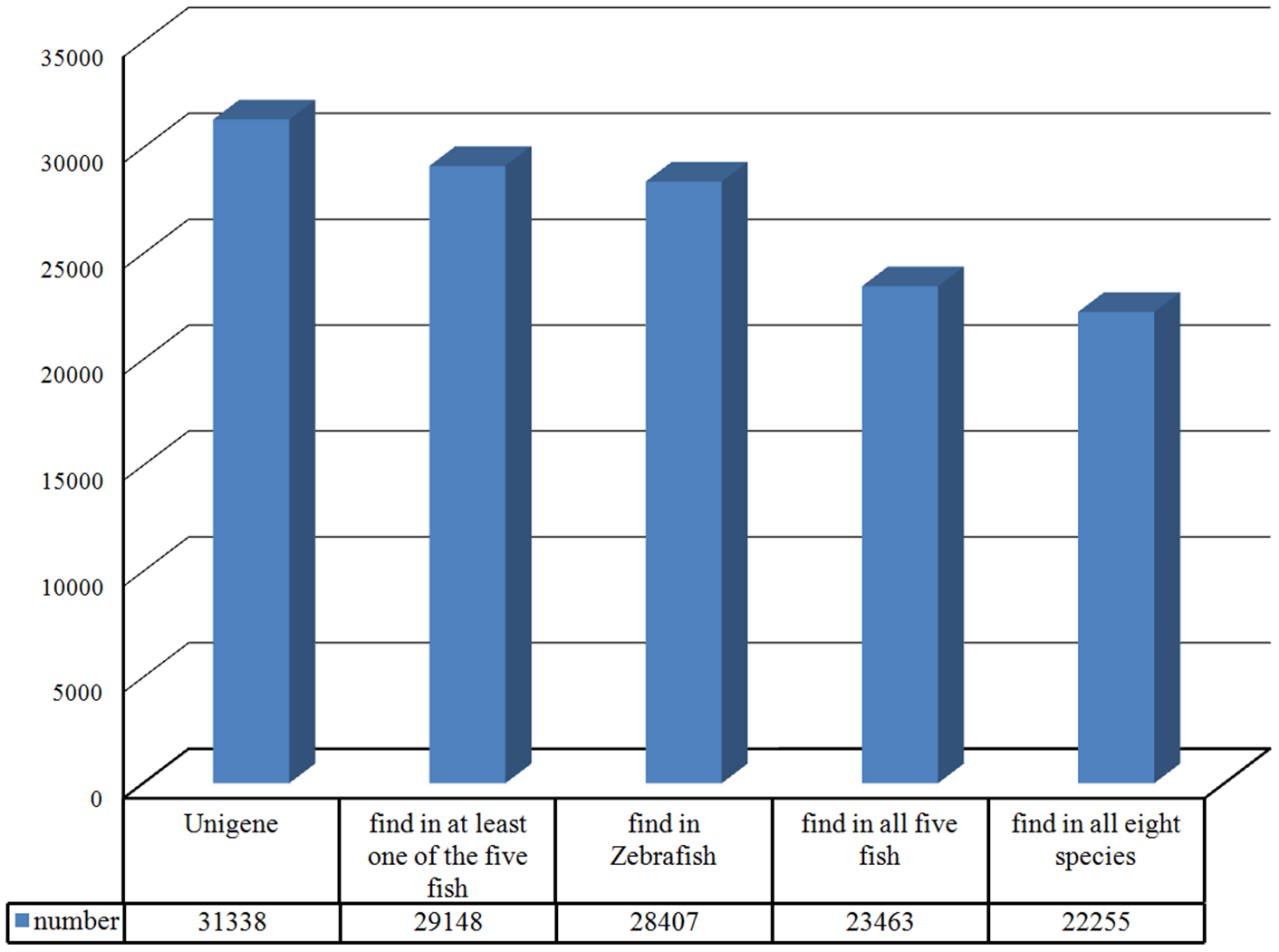
Genes conserved in blunt snout bream and five model fish species (zebrafish, medaka, *Tetraodon*, fugu, and stickleback), humans, mice, and chickens. Unigenes of the blunt snout bream were characterized by searching against public databases. The number of blunt snout bream homologous genes identified in other species by using BLASTX.

Characterization of blunt snout bream unigenes by searching against public databases: E-value distribution of the top hits in the databases showed 72% matched sequences with a strong homology (<1.0e-50) (Fig 1a); 46% of the transcripts had a similarity higher than 80%, while 32% showed a similarity between 60% and 80% with respect to the identity distribution pattern. Therefore, 78% of the transcripts showing an identity higher than 60% along with a high-quality e-value distribution supported the reliability of the *de novo* assembly (Fig 1b). To assess the evolutionary conservation of the identified unigenes, the number of hits to the number of unigenes in zebrafish, medaka, *Tetraodon*, fugu, stickleback, humans, mice, and chickens was compared (Table 4). A total of 22,255 (71%) putative known unigenes were found in all eight species; 23,463 (74.9%) were found in all five fish species; 28,407 (90.6%) were found in zebrafish; and 29,148 (93.1%) were found in at least one of the five fish species (Fig 2), indicating a high level of conservation of gene content among the blunt snout bream and other species, especially teleost fish species [46].

**Table 4:** Summary of Blast X search analysis of unique sequences of blunt snout bream.

Refseq/Ensembl	Blunt snout bream hits*	Unique protein	Percentage of total unique proteins
Zebrafish	28,407	21,107	48.91% of 43,153
Medaka	25,822	15,059	61.03% of 24,674
Tetraodon	25,145	14,693	63.56% of 23,118
Fugu	25,521	17,495	36.57% of 47,841
Stickleback	25,934	16,063	58.25% of 27,576
Human	25,228	16,702	16.79% of 99,459
Mouse	25,168	15,268	28.81% of 52,998
Chicken	24,332	11,869	72.58% of 16,354

Note:

*Number of significant (E-value <1e-10) alignments using all blunt snout bream unique sequences as queries to search the listed databases.

### Annotation and classification

A total of 31,338 unigenes were assigned to 104 functional groups with 143,964 functional terms (S2 Fig). For the three main categories of the GO classification scheme, assignments to biological processes (69,454, 48%) made up the majority, followed by the cellular components (48,039, 33%) and molecular functions (26,471, 18%). Among these GO groups, a high number of unigenes putatively involved in molecular functions (12,289) and biological processes (10,843) indicated that the blunt snout bream tissues used in this study underwent unique metabolic activities related to growth and tolerance to hypoxia, which coincided with the status of the samples. Under the category of cellular components, the cell (10,117), intracellular component (8,996), cellular component (8,736), organelle (6,746), and cytoplasm (5,131) were prominent groups (S2 Fig).

**Fig 3:**
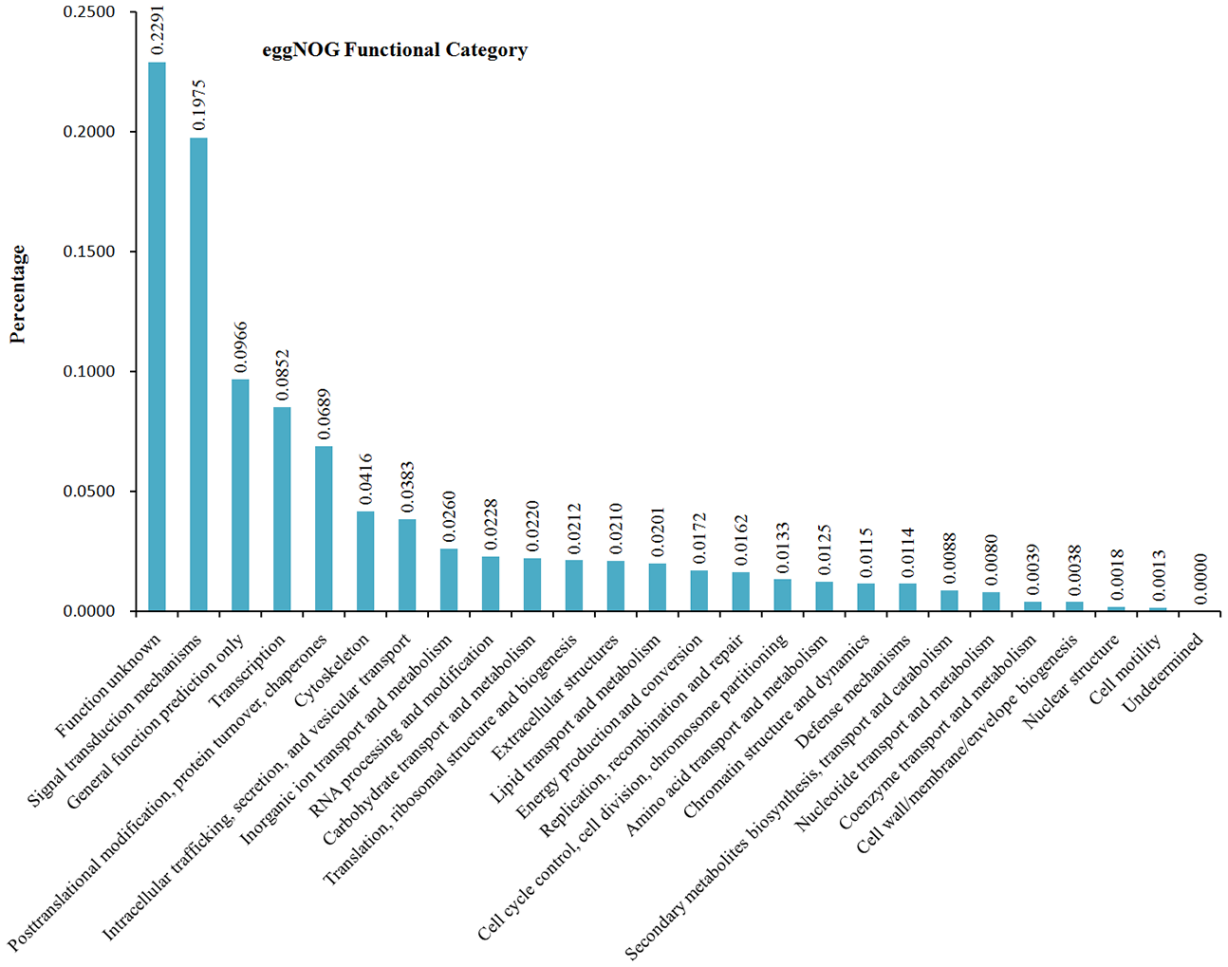
eggNOG functional categories.

On the basis of the literature [47], 31,338 unigenes could also be classified into 25 eggNOG categories (Fig 3). 22.91% unigenes (7,761) were assigned to the unknown functional group, which might indicate an unknown mechanism underlying growth and oxygen resistance in the blunt snout bream. Signal transduction mechanisms (6,691, 19.75%) were the second largest functional group, which indicated that the mechanisms are mostly related to environmental information processing. The third largest functional group was general function prediction only (3,272, 9.66%), which was consistent with the transcriptome results from other studies [48–53]. The relative abundant groups were transcription (2,887, 8.52%), post-translational modification, protein turnover, chaperones (2,334, 6.89%), cytoskeleton (1,410, 4.16%), intracellular trafficking, secretion, and vesicular transport (1,298, 3.83%); the two groups involving cell motility and nuclear structures comprised a total of 105 unigenes (0.31%), representing the smallest eggNOG classifications. It is noteworthy that 0.88% unigenes (299) were classified into the secondary metabolite biosynthesis group (Fig 3).

**Fig 4:**
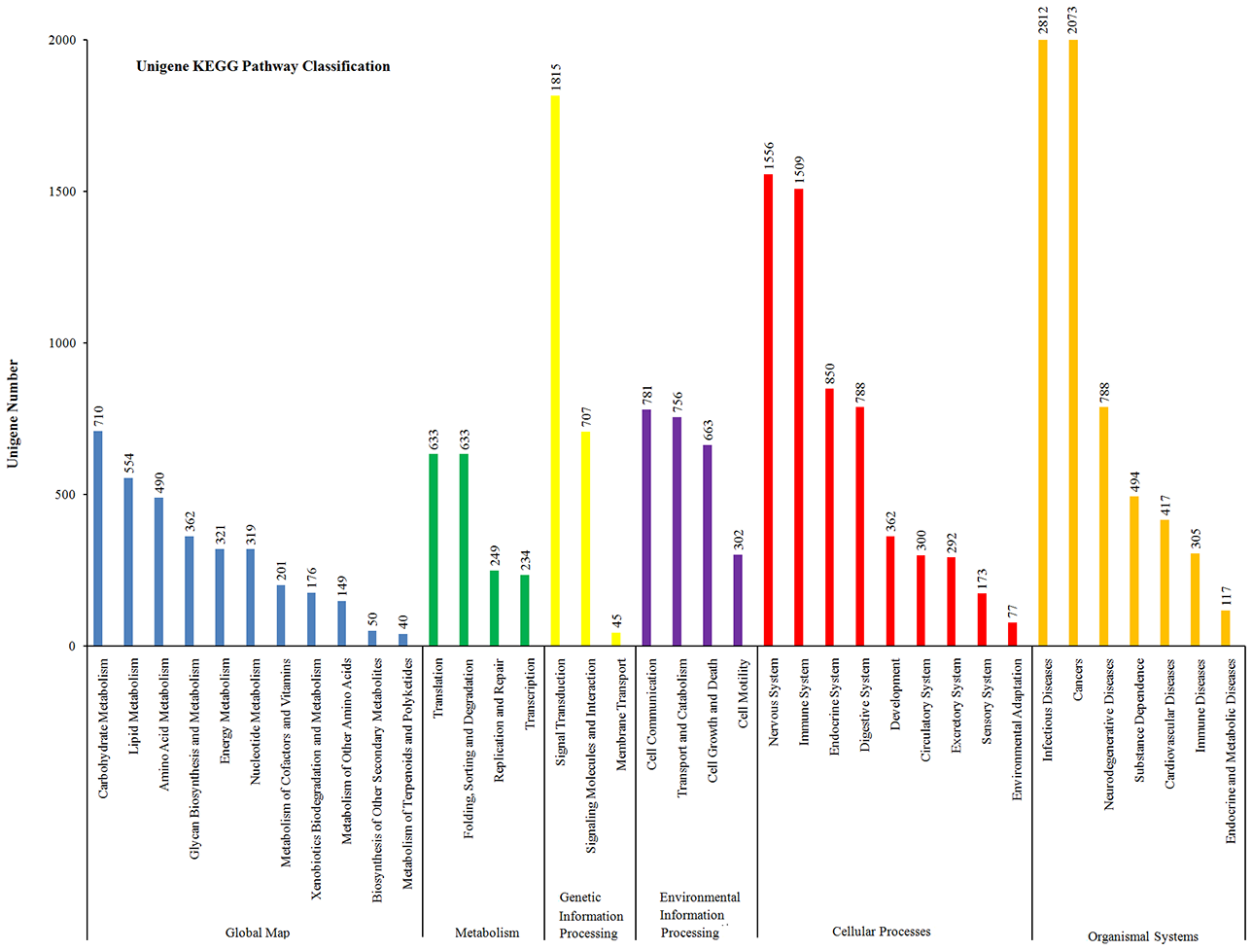
Classification of unigenes on the basis of KEGG categorization.

Functional classification of KEGG provided a valuable resource for investigating specific processes and pathways in the liver and gills of the blunt snout bream. A total of 23,103 unigenes were classified into seven main categories, including 45 secondary pathways in the eight tested samples (Fig 4). Human diseases was the largest category (7,006, 30.33%), followed by organismal systems (5,907, 25.57%), metabolism (3,372, 14.6%), environmental information processing (2,567, 11.11%), and cellular processes (2,502, 10.82%); genetic information processing (1,749, 7.57%) was the smallest category (Fig 4). These results indicate that active metabolic and genetic processes affected by dissolved oxygen levels occur in the liver and gills of the blunt snout bream.

### Gene expression patterns and pathway classification enrichment analysis

**Fig 5:**
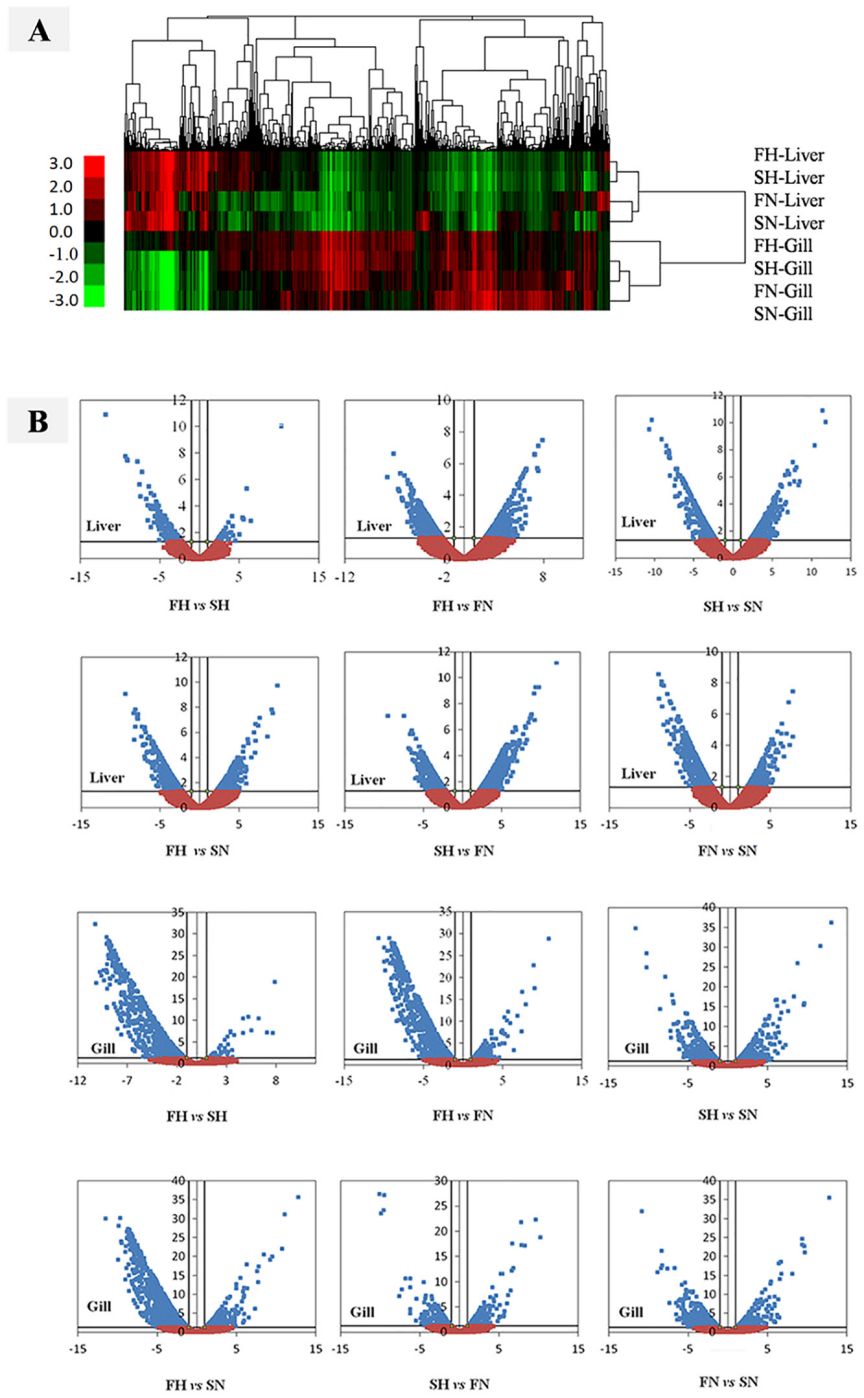
Analysis of differentially expressed genes in the blunt snout bream. (A) Clusters of differentially expressed genes in the liver and gills of the blunt snout bream from four groups, as shown in Table 1. Expression changes and cluster analysis of 6,639 genes that were differentially expressed between any two tissue samples. Each column represents a differentially expressed gene, and each row represents a sample. Changes in expression levels are shown using color scales with saturation at >2-fold changes. Green and red gradients indicate a decrease and increase in transcript abundance, respectively. Clustering was performed using the Pearson distance method, and hierarchical clustering, pairwise centroid–linkage. (B) DE gene analysis and volcano plot for 12 comparative groups. The x-axis is the value of *Log2* (Fold Change), and the y-axis is the value of–*Log10* (*p*-value). Vertical lines are boundaries of difference threshold of 2-fold change, and the horizontal line is *p<*0.05. The blue dots present differential expression genes that meet the requirements, while the orange dots present differential expression genes that do not meet the requirements.

Gene expression patterns can provide important clues as to the roles of unknown genes in biologically active processes [54]. While reads per kilobase per million mapped reads (RPKM) are widely used to calculate the gene expression value [55], we used a more accurate DESeq method to estimate gene expression values in order to infer differential expression signals with good statistical power [37]. To identify DE genes among the eight samples, we compared the samples and selected a total of 6,639 unigenes, which were at least 2-fold up- or down-regulated with a *p*-value < 0.05. Then, hierarchical clusters were generated to obtain a global view of the DE genes (Fig 5A). The genes detected in the different groups were clearly separated, and an increase or decrease in transcript abundance in the liver was different from that in the gills from the blunt snout bream. Liver-FH showed a closer relationship with Liver-SH, while Liver-FN showed a closer relationship with Liver-SN; and the former two showed a greater distance than the latter two (Fig 5A). However, Gill-SH showed a closer relationship with Gill-FN, and they were closer to Gill-SN than to Gill-FH. These results further prove that the diversity in hypoxia response between the fast- and slow-growth families was regulated by genes, and that the gill samples showed a higher amount of DE genes resulting from physiological response of hypoxia adaption. This also indicates that the gill is actually a sensitive and important organ response to hypoxic stress.

With simultaneous display of two correlated pieces of information (fold change and *p*-value), volcano plots are commonly used in the microarray analysis of mRNA expression levels [56–59]. The abundance of blue dots shows the number of DE genes, and their locations with respect to vertical lines reveal the up-regulation of DE genes in each comparative group (Fig 5B). In view of different groups, up-regulation expression accumulate to 5,608 (Gill-FH 3,826, Liver-FH 1,782), 3,625 (Gill-SN 2,227, Liver-SN 1,398), 3,519 (Gill-SH 1,561, Liver-SH 1,958), 3,047 (Gill-FN 1,172, Liver-FN 1,875). In summary, 13,887 up-regulation DE genes were detected, including 8,097 in gill and 5,790 in liver of four groups.

**Fig 6:**
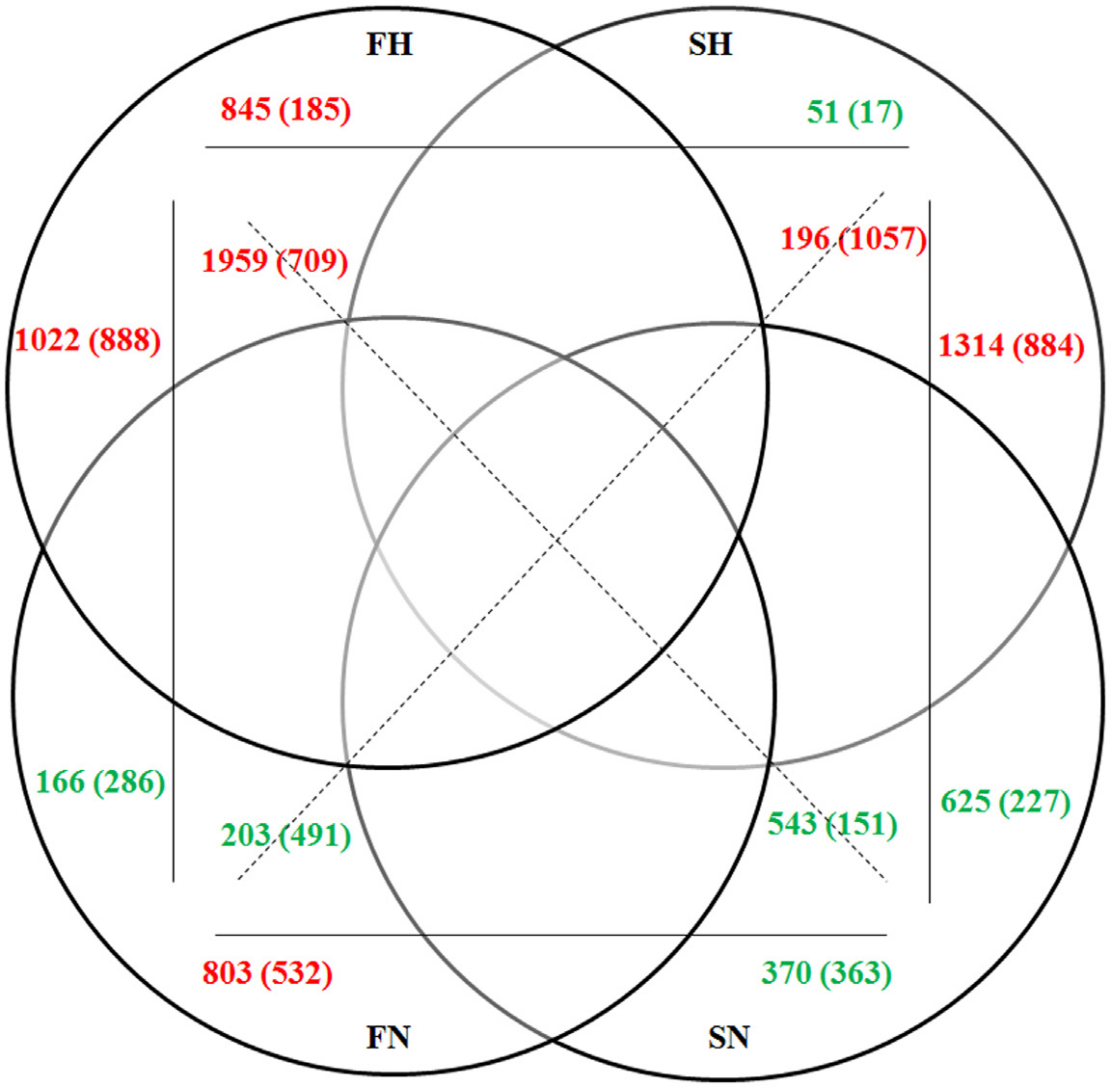
Venn diagrams showing DE genes up-regulated and down-regulated in the gills and liver. All these differential genes were determined by pair-wise comparison (>2-fold change, *p <* 0.05, RPKM > 3). Numbers appeared in horizontal, vertical and diagonal direction show up- (red numbers) or down- (green numbers) regulated DE genes between both groups. The numbers outside and within the brackets show the DE genes of gills (outside) and liver (within), respectively.

Venn diagram analysis of DE unigenes in comparative groups [>2-fold change, *p* < 0.05, RPKM > 3) also yielded the same results. There were more up-regulated DE unigenes in both F and S under conditions of hypoxia rather than normoxia (Fig 6). Additionally, the number of up-regulated or down-regulated DE unigenes was higher in F than in S, regardless of hypoxia or normoxia, in both the liver and gills (Fig 6). These results suggest that the pattern of increased transcript abundance along with hypoxic stress was an important response mechanism for low dissolved oxygen levels. In addition, this indicates that the fast-growth family may have more positively regulated genes than negatively regulated ones in the hypoxia stress response pathway.

### Expression analysis of putative DE genes related to hypoxia and growth

**Fig 7:**
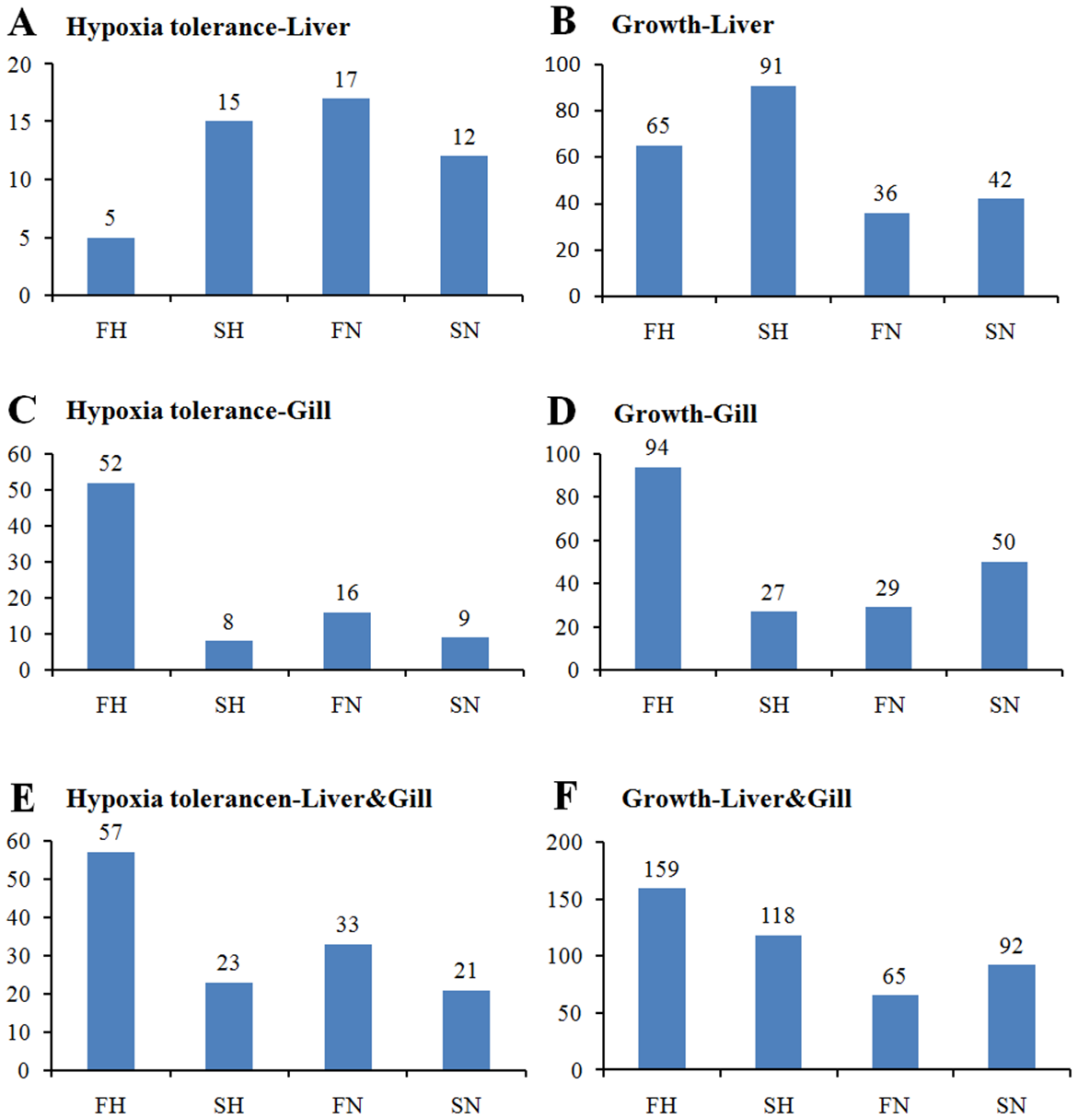
Expression analysis of putative DE genes related to hypoxia response and growth in different samples. (A, B) Putative DE genes related to hypoxia response (A) and growth (B) in the liver; (C, D) Putative DE genes related to hypoxia response (C) and growth (D) in the gills; (E, F) Total number of putative DE genes related to hypoxia response (E) and growth (F) in both the liver and gills.

To date, there have been few studies on the hypoxia response mechanism in the blunt snout bream. In this study, DE gene expressions in the liver and gills of four groups were compared pair-wise, and genes related to growth and hypoxia response were screened. Expression abundance of the genes showed a significant difference. Putative DE genes related to hypoxia response in Liver-FN (17) were expressed to a greater extent than those in Liver-SH (15), Liver-SN (12), and Liver-FH (5), while putative DE genes in Gill-FH (52) were expressed to a greater extent than those in Gill-SH (8), Gill-FN (16), and Gill-SN (9) (Fig 7). The total number of DE genes related to hypoxia response pathways in the four groups was in the order of FH (57) > FN (33) > SH (23) > SN (21). The putative DE genes related to hypoxia response can mainly be classified as: hypoxia-inducible factor (*HIF-1α*, *HIF-2α*, *HIF-3α*) and its co-transcription factors (eg. *Arnt*, *Ncoa1* and *Per 1*), *HIF-1α* interactors (eg. *Apex1*, *Egln1*, *Tp53*, *PHDs*, *pVHL*, *FIH*,), responsive genes including angiogenesis (eg.*EGR1*, *EDN1*, *EPO*, *Hmox1*, *PGF*, *VEGFa*), coagulation (eg. *ALDOA*, *SLC16A3*), DNA damage signaling and repair (eg. *ATR*), metabolism (eg. *ENO1*, *ERO1L*), regulation of apoptosis (eg. *eNOS*), regulation of cell proliferation (eg. *EGR1*, *IGFBP3*), transcription factors (eg. *FOS*), transporters, channel and receptors (*SLC2A1*, *SLC16A3*), other responsive genes (eg. *MAP3K1*), and pathway activity signature genes. As shown in Fig 7, the total number of DE genes related to growth pathways was in the order of FH (159) > SH (118) > SN (92) > FN (65). The DE genes related to growth pathways were mainly classed as: growth factors and receptors (eg. *GHRH*,*VEGFA*, *VEGFR1*, *VEGFR2*, *PDGFR*, *IGF1R*, *EFG*, *EFGR*, *TGF-β*), binding protein (eg. *IGFBP1*, *BMP*), metabolism enzymes (eg. Glycogen debranching enzyme, Lacate dehydrogenase, Acyl-CoA synthase, Glycerol-3-phosphate dehydrogenase, malic dehydrogenase), and pathway activity signature genes (*Ras*, *PI3K*, *mTOR*, *PKB*, *PKC*).

### Regulatory network of hypoxia signaling pathway and growth-related pathways

By comparing the up-regulation of DE gene expressions in the liver and gills of FH and SH groups, we obtained 17 unigenes related to growth (eg. *GHR 1/2*, *EGR2/3*, *IGF1*, *IGF1R*, *FGF*, *FGFR*, *EGFR*, *FGFR4*, *EGF*, *FRS2*, *GADD45*, *HBEGF*, *IGFBP3*, *TGFB1/2/3*, *GFBR1/2*, *VEGFA/B*, *VEGFC/D*), matched 54 sequences, and mapped 20 KO pathways (S3 Table). By comparing the up-regulation of DE gene expressions in the liver and gills of FH and FN, we obtained 26 unigenes or enzymes related to hypoxia response (eg. *HIF1A*, *HIF2A*, *EPOR*, *EPO*, *CYP7A1*, *CYP27A*, etc.), matched 64 sequences, and mapped 26 KO pathways (S3 Table). Our results show that growth inhibition under hypoxic stress may be due to the expressions of these putative DE genes; thus, selective breeding under hypoxic conditions may be an effective and direct breeding method.

The up-regulated DE genes in the fast-growth family under hypoxic stress indicated that these genes would be involved in different physiological functions against hypoxic stimulations. For instance, the hypoxia-induced factor (HIF) and its co-transcription factors are of the master regulator in the hypoxia signaling pathway, which widely affect glucose metabolism, cell proliferation, apoptosis, angiogenesis, hypoxic acclimatization, embryonic development, various ischemic diseases and tumorigenesis [60–61]. Under hypoxic condition, the body weight of the fast-growth family at 120-day was 15.68 g, which was 1.86 times as heavy as that (8.43 g) of the slow-growth family of blunt snout bream (Table 1). Therefore, some unique molecular mechanisms might have been developed as adaptive strategies to cope with hypoxia in the fast-growth family [7, 62]. Moreover, the body weight of the fast-growth group under hypoxic treatment condition was only 55% of weight (28.33 g) cultured under normoxic condition. This implied that fast-growth group may sacrifice some growth in order to survive in a hypoxic environment. Consistently with our previous data in blunt snout bream as well as in grass carp, hypoxia treatment can induce significant embryonic developmental delay and growth retardation by inhibiting related insulin growth factor (IGF) signaling pathway [29, 36]. As these DE genes related to hypoxia and growth pathways belongs to different complex gene network, further studies are needed to explore the mutual regulatory mechanisms between them.

### Validation of RNA-seq data by qRT-PCR

**Fig 8:**
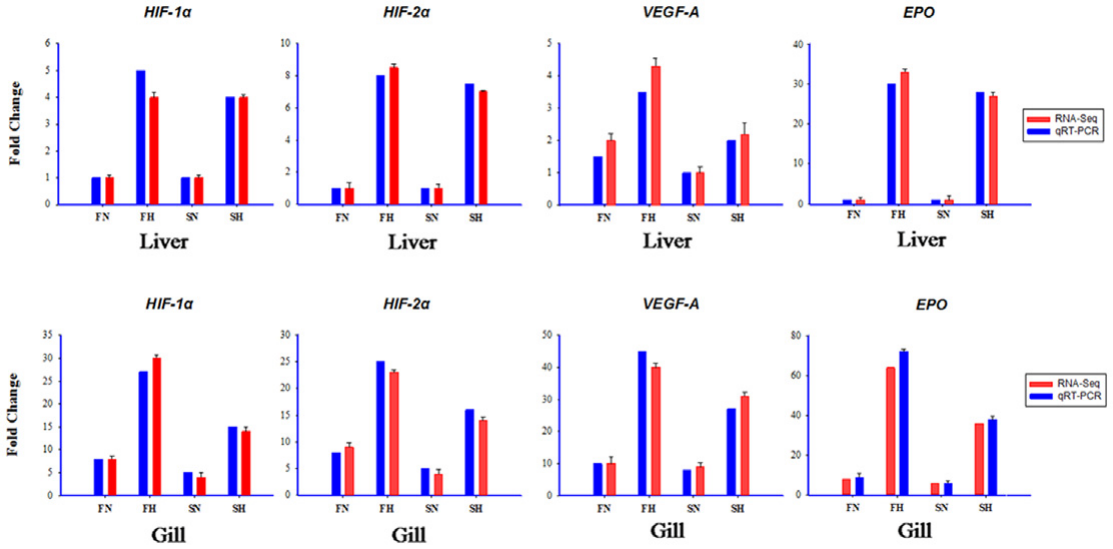
Validation of RNA-seq data by using quantitative real-time polymerase chain reaction (qRT-PCR). Expressions of *HIF-1α*, *HIF-2α*, *VEGF-A*, and *EPO* were detected using RNA-seq (blue column) and qRT-PCR (red column). X-axis, group name; y-axis, fold change in gene expression.

The expression profiles of genes identified using Illumina sequencing were confirmed by measuring the relative mRNA levels of *HIF-1α*, *HIF-2α*, *VEGF-A*, and *EPO* related to hypoxia by using qRT-PCR (Fig 8). Our results indicate that the data of qRT-PCR were consistent with and validated those of RNA-seq.

## Conclusions

We performed *de novo* transcriptome sequencing of the liver and gill tissues from the fast- and slow-growth families of the blunt snout bream under hypoxic stress and normoxia. We obtained 31,338 unigenes and 6,639 DE genes (>2-fold change, *p* < 0.05, RPKM > 3). Expression of the DE genes was compared pair-wise, and number of genes related to the hypoxia response pathways in the four groups was found to be in the order of FH (57) > FN (33) > SH (23) > SN (21), while the number of genes related to growth pathways was in the order of FH (159) > SH (118) > SN (92) > FN(65). Moreover, qRT-PCR data for four DE genes (*HIF-1α*, *HIF-2α*, *VEGF-A*, and *EPO*) were consistent with and validated the RNA-seq data. Growth performance of the fast-growth family under hypoxic stress may be due to the expression levels of these differential genes. Therefore, some unique molecular mechanisms might have been developed as adaptive strategies to cope with hypoxia in the fast-growth family. Our study will not only lay a foundation for further studies on growth regulation under hypoxic conditions but also facilitate selective breeding for this important aquaculture species.

## Supporting Information

S1 Fig Length distribution of contigs (A), transcripts (B), and unigenes (C). (TIF)

S2 Fig GO analysis of unigenes. (TIF)

S1 Table Characterization of raw data. (DOCX)

S2 Table Characterization of clean data. (DOCX)

S3 Table Matched sequences and mapped KO pathways of genes or enzymes related to growth and hypoxia (DOC)

## Abbreviations

DE: differential expression genes
eggNOG: evolutionary genealogy of genes: Non-supervised Orthologous Groups
GO: Gene Ontology
KEGG: Kyoto Encyclopedia of Genes and Genomes
KO: KEGG Orthology
MS-222: tricaine methanesulfonate
NR: non-redundant database
qRT-PCR: quantitative real-time polymerase chain reaction
RPKM: reads per kilobase per million mapped reads
