# Supplementary figures and images for: Transcriptome Analysis of Blunt Snout Bream (Megalobrama amblycephala) Reveals Putative Differential Expression Genes Related to Growth and Hypoxia
Source: PLoS One. 2015 Nov 10;10(11):e0142801. doi: 10.1371/journal.pone.0142801 (PMC4640810; doi:10.1371/journal.pone.0142801)

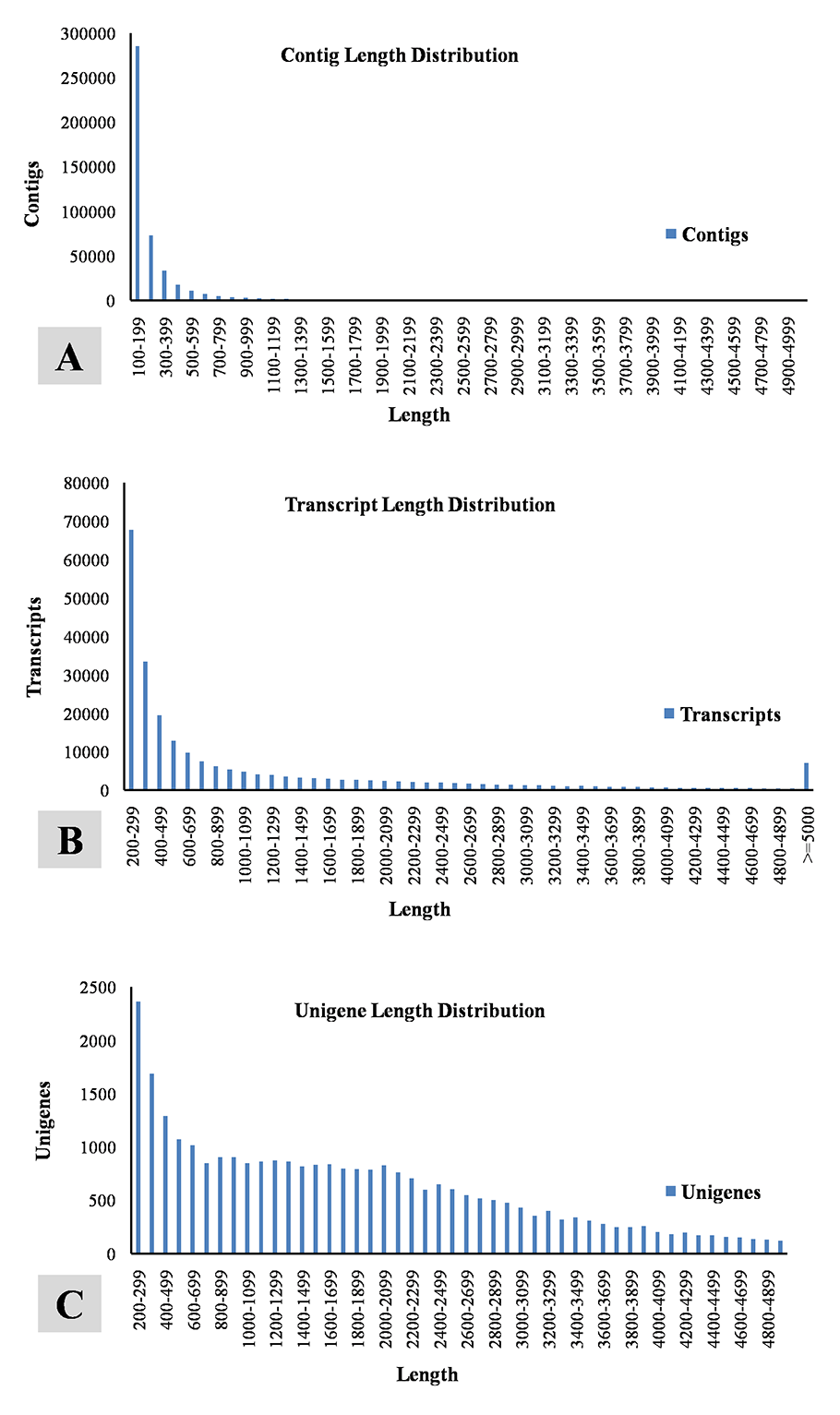

Supplement: S1 Fig — (TIF) [file pone.0142801.s001.tif]

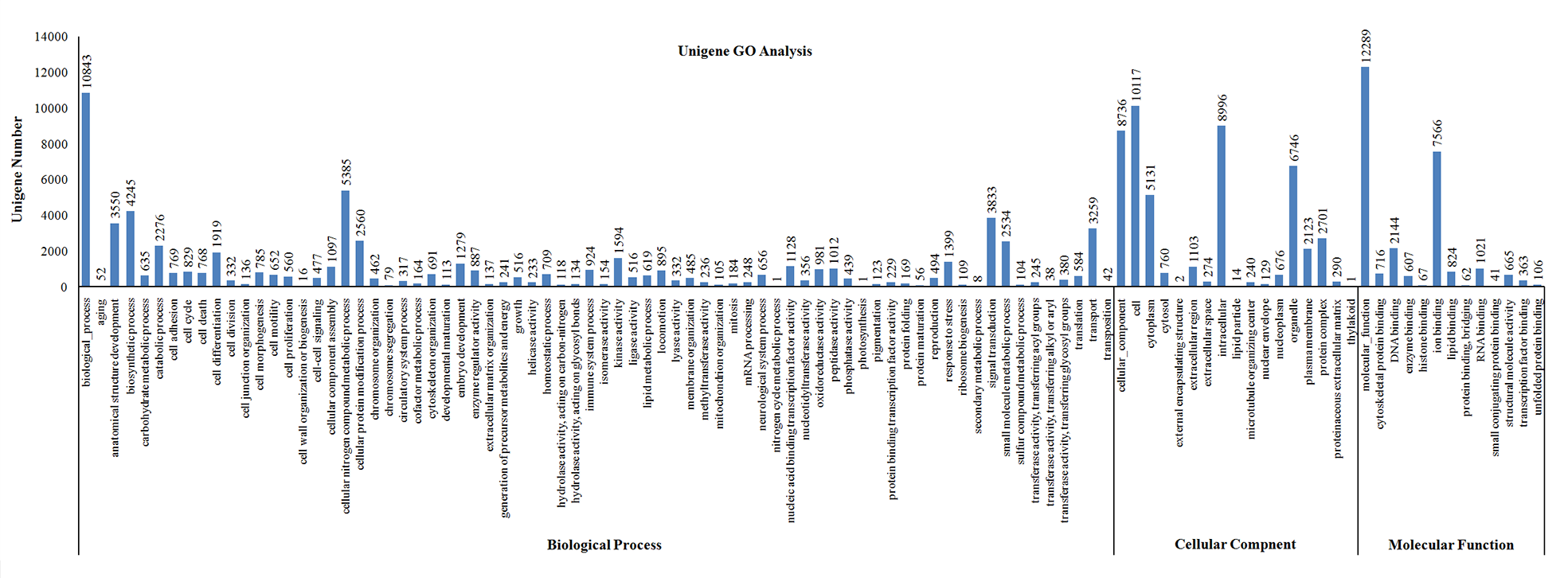

Supplement: S2 Fig — (TIF) [file pone.0142801.s002.tif]
