# Supplementary material for: Transcriptome Analysis of Blunt Snout Bream (Megalobrama amblycephala) Reveals Putative Differential Expression Genes Related to Growth and Hypoxia
Source: PLoS One. 2015 Nov 10;10(11):e0142801. doi: 10.1371/journal.pone.0142801 (PMC4640810; doi:10.1371/journal.pone.0142801)
Supplement: S1 Table — (DOCX) [file pone.0142801.s003.docx]

**Table S1. Characterization of raw data**

| **Sample** | **Reads** | **Raw Reads** | **Raw Data (bp)** | **Reads Length (bp)** |
| --- | --- | --- | --- | --- |
| Liver of FH | R1 | 22,957,545 | 2,295,754,500 | 100 |
|  | R2 | 22,957,545 | 2,295,754,500 |  |
|  | Paired | 22,957,545 | 4,591,509,000 |  |
| Gill of FH | R1 | 26,864,668 | 2,686,466,800 | 100 |
|  | R2 | 26,864,668 | 2,686,466,800 |  |
|  | Paired | 26,864,668 | 5,372,933,600 |  |
| Liver of SH | R1 | 24,650,760 | 2,465,076,000 | 100 |
|  | R2 | 24,650,760 | 2,465,076,000 |  |
|  | Paired | 24,650,760 | 4,930,152,000 |  |
| Gill of SH | R1 | 27,471,241 | 2,747,124,100 | 100 |
|  | R2 | 27,471,241 | 2,747,124,100 |  |
|  | Paired | 27,471,241 | 5,494,248,200 |  |
| Liver of FN | R1 | 31,589,909 | 3,158,990,900 | 100 |
|  | R2 | 31,589,909 | 3,158,990,900 |  |
|  | Paired | 31,589,909 | 6,317,981,800 |  |
| Gill of FN | R1 | 25,758,101 | 2,575,810,100 | 100 |
|  | R2 | 25,758,101 | 2,575,810,100 |  |
|  | Paired | 25,758,101 | 5,151,620,200 |  |
| Liver of SN | R1 | 23,001,238 | 2,300,123,800 | 100 |
|  | R2 | 23,001,238 | 2,300,123,800 |  |
|  | Paired | 23,001,238 | 4,600,247,600 |  |
| Gill of SN | R1 | 26,871,478 | 2,687,147,800 | 100 |
|  | R2 | 26,871,478 | 2,687,147,800 |  |
|  | Paired | 26,871,478 | 5,374,295,600 |  |
| Average | Paired | 26,145,618 | 5,229,123,500 |  |
| Total | Paired | 209,164,940 | 41,832,988,000 |  |
