# Supplementary material for: Transcriptome Analysis of Blunt Snout Bream (Megalobrama amblycephala) Reveals Putative Differential Expression Genes Related to Growth and Hypoxia
Source: PLoS One. 2015 Nov 10;10(11):e0142801. doi: 10.1371/journal.pone.0142801 (PMC4640810; doi:10.1371/journal.pone.0142801)
Supplement: S2 Table — (DOCX) [file pone.0142801.s004.docx]

**Table S2. Characterization of clean data**

| **Sample** | **Reads** | **Clean reads** | **Clean data (bp)** | **Useful reads %** | **Useful data %** |
| --- | --- | --- | --- | --- | --- |
| Liver of FH | R1 | 20,345,576 | 1,987,524,049 | 88.62% | 86.21% |
|  | R2 | 20,345,576 | 1,971,030,829 |  |  |
|  | Paired | 20,345,576 | 3,958,554,878 |  |  |
| Gill of FH | R1 | 23,797,104 | 2,324,254,497 | 88.58% | 86.16% |
|  | R2 | 23,797,104 | 2,304,964,584 |  |  |
|  | Paired | 23,797,104 | 4,629,219,081 |  |  |
| Liver of SH | R1 | 21,750,555 | 2,121,516,624 | 88.23% | 85.73% |
|  | R2 | 21,750,555 | 2,105,124,405 |  |  |
|  | Paired | 21,750,555 | 4,226,641,029 |  |  |
| Gill of SH | R1 | 24,270,976 | 2,369,705,485 | 88.35% | 85.92% |
|  | R2 | 24,270,976 | 2,350,934,832 |  |  |
|  | Paired | 24,270,976 | 4,720,640,317 |  |  |
| Liver of FN | R1 | 28,025,206 | 2,734,452,174 | 88.72% | 86.26% |
|  | R2 | 28,025,206 | 2,715,569,075 |  |  |
|  | Paired | 28,025,206 | 5,450,021,249 |  |  |
| Gill of FN | R1 | 22,697,378 | 2,215,426,291 | 88.12% | 85.67% |
|  | R2 | 22,697,378 | 2,198,116,737 |  |  |
|  | Paired | 22,697,378 | 4,413,543,028 |  |  |
| Liver of SN | R1 | 20,443,713 | 1,997,653,182 | 88.88% | 86.51% |
|  | R2 | 20,443,713 | 1,982,182,577 |  |  |
|  | Paired | 20,443,713 | 3,979,835,759 |  |  |
| Gill of SN | R1 | 23,730,130 | 2,317,167,233 | 88.31% | 85.89% |
|  | R2 | 23,730,130 | 2,298,963,090 |  |  |
|  | Paired | 23,730,130 | 4,616,130,323 |  |  |
|  |  |  |  |  |  |
|  |  |  |  |  |  |
|  |  |  |  |  |  |
|  |  |  |  |  |  |
|  |  |  |  |  |  |
| Average | Paired | 23,132,580 | 4,499,323,208 |  | |
| Total | Paired | 185,060,638 | 35,994,585,664 |  | |
