# Supplementary material for: Transcriptome Analysis of Blunt Snout Bream (Megalobrama amblycephala) Reveals Putative Differential Expression Genes Related to Growth and Hypoxia
Source: PLoS One. 2015 Nov 10;10(11):e0142801. doi: 10.1371/journal.pone.0142801 (PMC4640810; doi:10.1371/journal.pone.0142801)
Supplement: S3 Table — (DOC) [file pone.0142801.s005.doc]

**S3 Table. Matched sequences and mapped KO pathways of genes or enzymes related to growth and hypoxia**

| Growth related (FH *v.s.* SH) | | |
| --- | --- | --- |
| Genes/Enzymes (17) | Sequences (54) | KO (20) |
| *GHR 1/2* | 2 | ko:K05080 |
| *EGR2/3* | 5 | ko:K12496 |
| *IGF1* | 4 | ko:K05459 |
| *IGF1R* | 6 | ko:K05087 |
| *FGF* | 4 | ko:K04358 |
| *FGFR* | 4 | ko:K04357 |
| *EGFR* | 4 | ko:K04361 |
| *FGFR4* | 2 | ko:K05095 |
| *EGF* | 4 | ko:K04357 |
| *FRS2* | 1 | ko:K12461 |
| *GADD45* | 2 | ko:K04402 |
| *HBEGF* | 1 | ko:K08523 |
| *IGFBP3* | 1 | ko:K10138 |
| *TGFB1/2/3* | 4 | ko:K13375 / ko:K13376 / ko:K13376 |
| *TGFBR1/2* | 6 | ko:K04387 / ko:K04388 |
| *VEGFA/B* | 2 | ko:K05448 |
| *VEGFC/D* | 2 | ko:K05449 |
| Hypoxia-response related (FH *v.s.* FN) | | |
| Genes/Enzymes (26) | Sequences (64) | KO (26) |
| *E1.14.11.1* | 2 | ko:K00471 |
| *E1.14.13.72* | 2 | ko:K07750 |
| *E1.14.13.8* | 2 | ko:K00485 |
| *E1.14.13.9* | 2 | ko:K00486 |
| *E1.13.11.11* | 2 | ko:K00453 |
| *HPD* | 3 | ko:K00457 |
| *BCMO1* | 2 | ko:K00515 |
| *HMOX* | 3 | ko:K00510 |
| *SQLE* | 2 | ko:K00511 |
| *KRAS* | 2 | ko:K07827 |
| *BAD* | 2 | ko:K02158 |
| *HIF1A* | 4 | ko:K08268 |
| *HIF2A* | 4 | ko:K09095 |
| *EPOR* | 2 | ko:K05079 |
| *EPO* | 3 | ko:K08726 |
| *CYP7A1* | 2 | ko:K00489 |
| *CYP27A* | 2 | ko:K00488 |
| *EPHX1* | 4 | ko:K01253 |
| *AKT* | 4 | ko:K04456 |
| *PIK3R* | 3 | ko:K02649 |
| *PTGS2* | 2 | ko:K11987 |
| *PLA2G4* | 2 | ko:K16342 |
| *CDO1* | 2 | ko:K00456 |
| *PLOD2* | 2 | ko:K13645 |
| *KRAS* | 2 | ko:K07827 |
| *RAC1* | 2 | ko:K04392 |
